# Effect of high-intensity interval training on aerobic capacity, physical fitness, and body composition in martial arts athletes: a systematic review and meta-analysis of randomized controlled trials

**DOI:** 10.3389/fnut.2026.1792680

**Published:** 2026-03-31

**Authors:** Mengbiao Cai, Haihua Cai, Chao Liu, Kai Qi

**Affiliations:** 1 Department of Physical Education, Nanjing University of Aeronautics and Astronautics, Nanjing, China; 2 Tianjin Municipal Bureau of Sports, Tianjin, China; 3 Nanjing Foreign Language School Hexi Junior Campus, Nanjing, China; 4 Gdansk University of Physical Education and Sport, Gdańsk, Poland

**Keywords:** aerobic capacity, athletes, athletic performance, body composition, high-intensity interval training, martial arts

## Abstract

**Objective:**

This meta-analysis aimed to evaluate the effects of high-intensity interval training (HIIT) on aerobic capacity, athletic performance, and body composition in martial arts athletes.

**Methods:**

A systematic search was conducted in multiple databases, including PubMed, MEDLINE, Web of Science, CNKI, ProQuest and SciELO, following PRISMA guidelines. Fourteen randomized controlled trials (RCTs) involving 348 participants were included. The inclusion criteria focused on martial arts athletes undergoing HIIT, with outcome measures including aerobic capacity (HRmax, VO_2_max), athletic performance (strength, agility, speed), and body composition (muscle mass, body fat percentage, body weight). Data were analyzed using a random-effects model to calculate standardized mean differences (SMD).

**Results:**

The findings demonstrated that HIIT significantly improved aerobic capacity, athletic performance, and body composition in martial arts athletes. Improvements in aerobic capacity were primarily reflected in VO_2_max (*SMD* = 1.04, 95% CI [0.74, 1.33], *I*^2^ = 16.3%, *p* = 0.284). With respect to athletic performance, significant improvements were observed in lower-limb muscular power (*SMD* = 0.33, 95% CI [0.02, 0.64], *I*^2^ = 48%, *p* = 0.017), agility (*SMD* = −0.45, 95% CI [−0.79, −0.11], *I*^2^ = 0%, *p* = 0.922), and speed (*SMD* = −0.45, 95% CI [−0.79, −0.11], I^2^ = 0%, *p* = 0.922). Regarding body composition, the primary improvement was observed in percent body fat (*SMD* = −0.51, 95% CI [−0.92, −0.09], *I*^2^ = 0%, *p* = 1.000). However, no significant improvements were found in HRmax, skeletal muscle mass, or body weight.

**Conclusion:**

HIIT represents an effective training modality for martial arts athletes, and future studies should focus on long-term, large-sample randomized controlled trials to further clarify differential effects across subgroups and training modalities, as well as to explore optimal strategies for integrating HIIT with martial arts–specific training.

## 1 Introduction

High-Intensity Interval Training (HIIT), characterized by the alternation of high-intensity exercise and intermittent recovery, has demonstrated substantial benefits across multiple domains, including public health promotion, chronic disease prevention, and enhancement of physical fitness (1–4). The primary physiological advantage of HIIT lies in its ability to simultaneously stimulate aerobic and anaerobic metabolic systems within a relatively short period, thereby improving cardiorespiratory endurance, muscular function, and metabolic health in parallel. Evidence indicates that well-designed HIIT protocols can significantly enhance maximal oxygen uptake (VO_2_max), muscular strength, and body composition. Improvements in VO_2_max are particularly important for martial arts athletes, as aerobic endurance is crucial for maintaining performance during sustained offense and defense transitions. HRmax, or the maximum heart rate, reflects the athlete's cardiovascular fitness, which is essential for recovery capacity between repeated bouts of high-intensity efforts, a common demand in combat sports. Moreover, HIIT has been shown to optimize neuromuscular coordination and exercise economy, making it a time-efficient and broadly adaptable training strategy (5–7).

Martial arts, as a highly comprehensive competitive sport, places extremely stringent demands on athletes' multidimensional physical abilities, including aerobic endurance for sustained performance, anaerobic power for explosive attacks, and muscle strength and flexibility for complex technical movements. Aerobic capacity and HRmax improvements directly contribute to the athlete's ability to perform well in repeated, high-intensity efforts, which are central to the demands of combat sports. These abilities include anaerobic power for explosive attacks, aerobic endurance for sustained offense and defense transitions, dynamic balance and joint flexibility for complex technical movements, and core and limb strength for resistance to strikes and force generation (8–10). Furthermore, a lower body fat percentage and a higher muscle mass ratio have been confirmed to be closely related to martial artists' attack efficiency, defensive stability, and recovery ability (11). Martial arts include various combative events such as judo, wrestling, and taekwondo, all of which have extremely high demands on athletes' aerobic capacity and overall physical performance, making these factors crucial in determining an athlete's competitive level (12–14).

Although HIIT has been widely applied in cyclical or intermittent sports such as football, basketball, and swimming, and has shown positive effects on athletes' overall physical fitness, its applicability and mechanisms in martial arts, which have complex technical structures and diverse energy metabolism patterns, remain to be explored (15–19). Current research on HIIT has found that most studies focus on the general population or specific disease groups (20, 21), and empirical studies on martial arts athletes are still relatively limited, with issues such as varying intervention designs, inconsistent evaluation metrics, and unclear long-term training effects.

Based on this, the present study employs a systematic review and meta-analysis approach to comprehensively assess the impact of HIIT on the aerobic capacity, physical fitness, and body composition of martial arts athletes in the existing literature. Through systematic review and quantitative synthesis, this study aims to provide evidence-based support for developing specialized and individualized HIIT intervention programs for martial arts, further promoting the scientific development of martial arts training, and offering theoretical guidance and practical insights for the design of interval training in other skill-based, physically demanding sports.

## 2 Materials and methods

This study conducted a systematic review and meta-analysis of randomized controlled trials (RCTs) to assess the impact of high-intensity interval training on aerobic capacity, athletic performance, and body composition in martial artists. This review was performed in accordance with the Preferred Reporting Items for Systematic Reviews and Meta-Analyses (PRISMA) (22) guidelines and preregistered in the PROSPERO database (ID: CRD420261278272).

### 2.1 Search strategy

We searched three English international databases (PubMed, MEDLINE, Web of Science), one Chinese database (CNKI), and two gray literature databases (ProQuest, SciELO), with the search period ranging from the establishment of the databases until December 28, 2025. We conducted a search using Medical Subject Headings (MeSH) for all relevant literature on HIIT and Martial Arts Athletes. Additionally, the search strategy utilized a combination of Boolean operators “AND” and “OR” to maximize the search results for relevant studies. To ensure comprehensive coverage, no time or language restrictions were applied. The detailed search method is provided in Supplementary Table S1.

### 2.2 Study selection

This systematic review and meta-analysis adhered to the guidelines established by the Cochrane Collaboration and the PRISMA framework (23). The study design was based on the PICOS principle, namely population, intervention, comparator, outcomes, and study design. The inclusion criteria of this study were based on the following research: (1) To enhance the robustness of the study, RCTs were included. (2) Participants were martial arts athletes, including those from disciplines such as karate, taekwondo, kung fu, judo, or mixed martial arts. (3) Experimental Group participants underwent HIIT as an intervention. (4) Studies must include a control group (e.g., no training, low-intensity training, or alternative exercise protocols) for comparison. (5) Both male and female athletes will be considered, without restriction based on gender. (6) Studies should report at least one of the following outcome measures: aerobic capacity (e.g., VO_2_max, HRmax), physical fitness (e.g., strength, agility, speed), or body composition (e.g., muscle mass, body fat percentage, weight). HRmax is defined as the maximum heart rate an individual can achieve during intense physical exertion, typically calculated as 220 minus the person's age. VO_2_max refers to the maximum rate of oxygen consumption measured during incremental exercise, and it is widely regarded as a key indicator of aerobic endurance.

This study followed the Cochrane Handbook guidelines and did not impose any language restrictions on the literature to maximize the inclusion of relevant research.

### 2.3 Extraction of data

Two review authors (C.M.B. and Q.K.) independently extract data using a Microsoft Excel template, which includes information such as the first author's name, publication year, participant characteristics, study design, intervention plan, and the mean and standard deviation of outcome measures before and after the intervention. Any discrepancies during the data extraction process were resolved through discussion and negotiation between the review authors to ensure consistency and accuracy. If the necessary information could not be obtained from the article, the review authors attempted to contact the corresponding author by email to inquire about the missing data. If essential key information remained unavailable, the study was excluded from the analysis. All data extraction and processing followed predefined standards to ensure the integrity and reliability of the data.

Additionally, in the fourteen studies included in this review, there are differences in the measurement dimensions, which need to be combined during the extraction process. As the data is presented in continuous variables, it needs to be converted according to the following formula: Allow the sample size of measurement method A to be N_1_, the mean to be M_1_ and the standard deviation to be SD_1_; the sample size of measurement method B to be N_2_, the mean to be M_2_ and the standard deviation to be SD_2_, then the combined sample size N = N_1_ + N_2_, the mean M=(N_1_M_1_+N_2_M_2_)/(N_1_+N_2_) and the standard deviation


$$\begin{array}{l} SD=\sqrt{\frac{\left(N_{1}-1\right)SD_{1}^{2}+\left(N_{2}-1\right)SD_{2}^{2}+\frac{N_{1}N_{2}}{N_{1}+N_{2}}\left(M_{1}^{2}+M_{2}^{2}-2M_{1}M_{2}\right)}{N_{1}+N_{2}-1}} \end{array}$$

If there were multiple dimensions of data to be combined, the data of those two dimensions would be consolidated first according to the above formula, then the obtained data were integrated with the third dimension, etc.

### 2.4 Methodological quality of included studies

This study used the Cochrane Risk of Bias assessment tool to evaluate the quality of the included studies, focusing on seven key areas: random sequence generation, allocation concealment, blinding of participants and researchers, blinding of outcome assessment, completeness of outcome data, selective reporting, and other potential sources of bias. According to the Cochrane Handbook for Systematic Reviews (24), the risk for each domain was be classified as low, high, or unclear. All studies were assessed by two independent reviewers, and any disagreements were resolved through discussion; if consensus could not be reached, arbitration was carried out through discussion to ensure objectivity and consistency in the assessment results. This assessment process ensured the comprehensive identification and control of bias risks, providing a safeguard for the final research quality.

### 2.5 Statistical analysis

This meta-analysis used Review Manager 5.4 and StataMP 15 for statistical analysis. During the data extraction process, we identified and adjusted for discrepancies in the measurement dimensions across the included studies. Since the data were presented as continuous variables, data transformation methods followed the relevant guidelines in the Cochrane Handbook for Systematic Reviews (6th edition). Specifically, for studies using different units or scales of measurement, we applied standardized methods to unify effect sizes to ensure comparability of the results (e.g., by appropriately weighting and merging means and standard deviations). The primary meta-analysis used a random-effects model, which is suitable for analyzing the expected variability between different populations, interventions, and measurement methods. This model accounts for both within-study and between-study variability, providing a more generalizable estimate of effects. We calculated the overall effect size (Z value), weighted mean difference (WMD), and their 95% confidence intervals (CIs). In addition, we used the *I*^2^ statistic to assess the presence of heterogeneity. If *I*^2^ > 50%, it was considered indicative of significant heterogeneity. In the presence of significant heterogeneity (*I*^2^ > 50%), subgroup or sensitivity analyses were performed to explore the sources of heterogeneity and assess the robustness of the results. Additionally, forest plots were generated to visually display publication bias across the included studies.

## 3 Result

### 3.1 Study identification and selection

**Figure 1:**
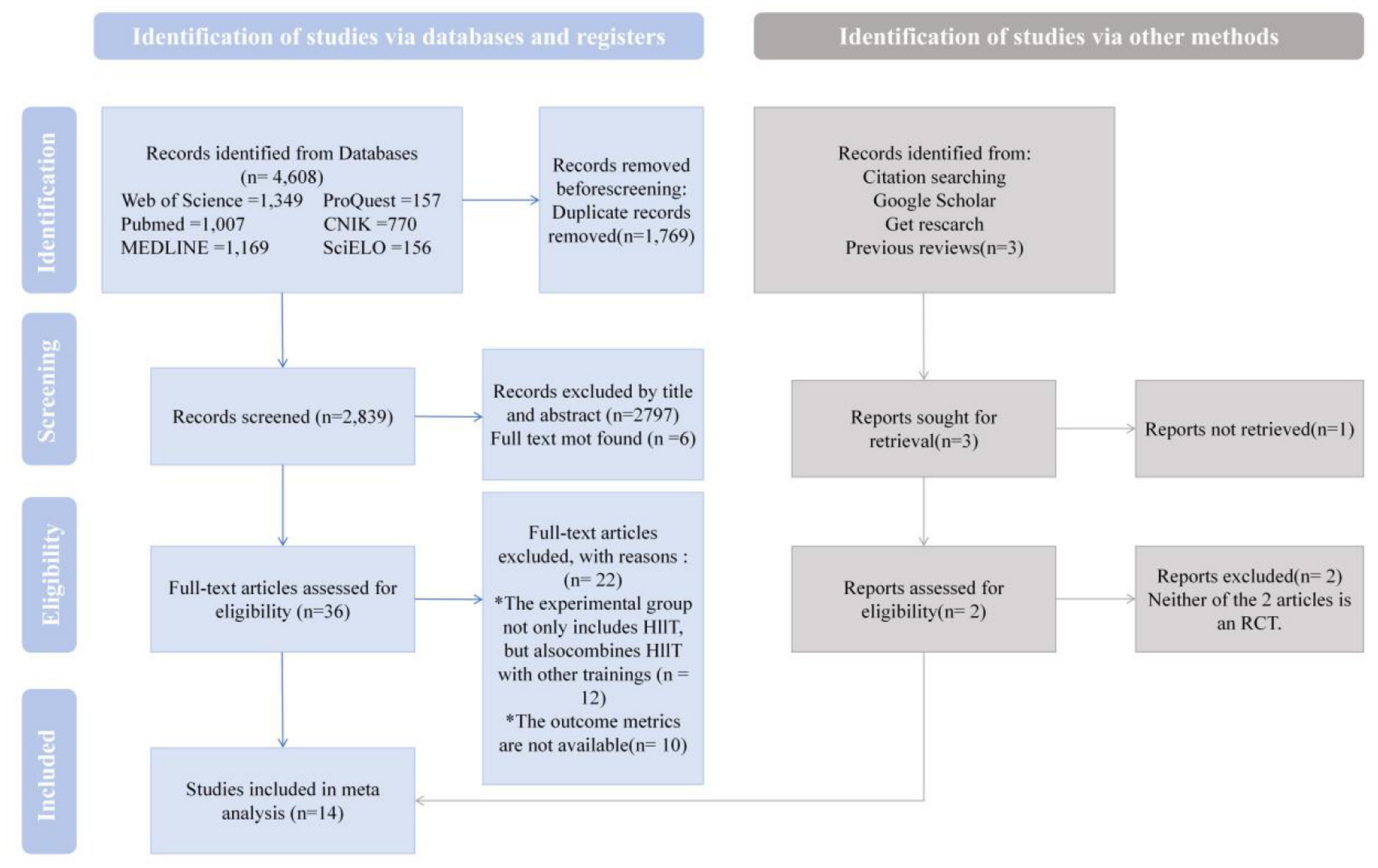
Flow chart diagram identifying the screening process and the studies included in the present review.

In this study, we systematically identified a total of 4,611 relevant articles, including 4,608 obtained through database searches and an additional three from other sources. After a stepwise screening process, 14 randomized controlled trials met the inclusion criteria and were incorporated into this meta-analysis (Figure 1).

### 3.2 Study characteristics

**Figure 2:**
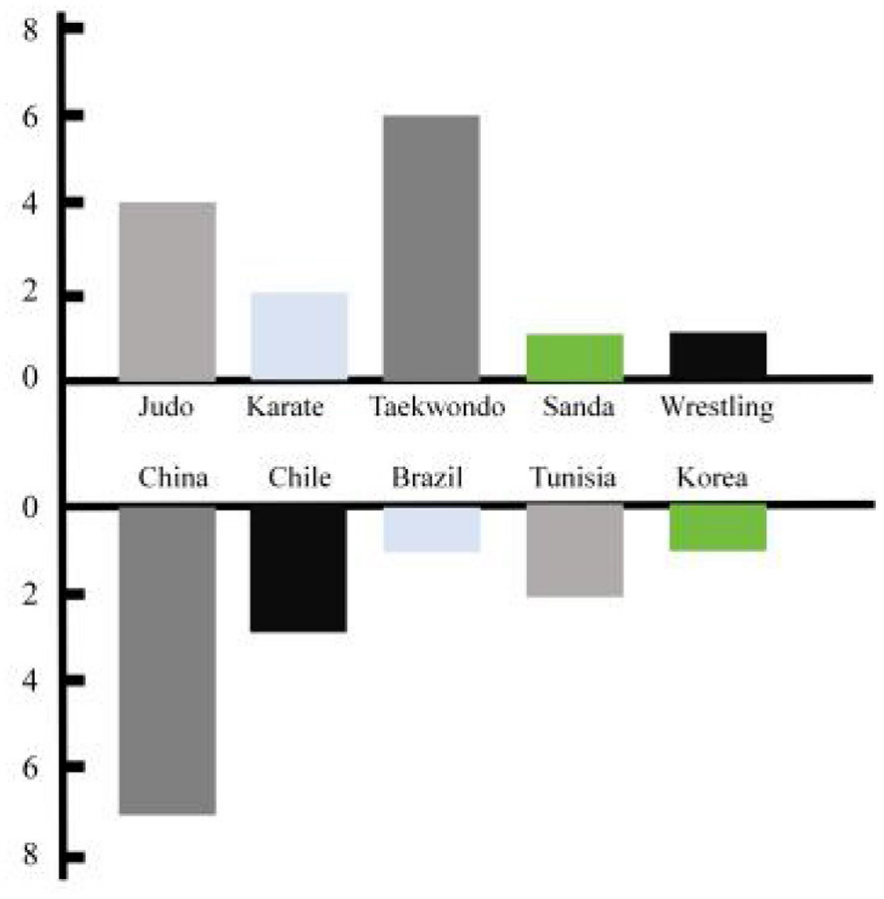
Research countries and Athletes information.

This study ultimately included 14 RCT studies, covering a total of 348 athletes (both male and female). The distribution of projects is as follows: four judo studies, two karate studies, six taekwondo studies, and one each on wrestling and sanda. These studies were mainly from countries such as China, Chile, Brazil, Tunisia, and Korea (see Figure 2 for details), with specific study characteristics and participant information provided in Table 1.

**Table 1:** Summary of study characteristics.

Study	Country; design	Participant	Training protocols		Details of HIIT intervention	Outcome indicators and measuring tools	Outcomes presented in the study
			Martial arts athletes	HIIT type			
Ho et al. (25)	China; RCT	*n* = 30; sex (male); EG1 (10) age = 20.64 ± 3.07 years; EG2 (10) age = 21.73 ± 6.15 years; CON (10) age = 21.55 ± 5.30 years competitive level: regional	judo	ITP	6 weeks, 2 days/week, the training consisted of two blocks, each containing six sets, with each set lasting 20 s, followed by a 20-s passive rest between sets. The total training duration was 8 min.	jump height (CMJ); PBF, SMM, BM (InBody 570 body composition analyzer); HRmax (polar H10 heart rate monitor)	jump height↑; PBF↓; SMM↑; BM↓; HRmax↓
Xu & Wang (26)	China; RCT	*n* = 24; sex (male); EG1 (8) age = 21.4 ± 2.2 years; EG2 (8) age = 21.7 ± 2.4 years; CON (8) age = 21.2 ± 2.4 years competitive level: national	karate	RT SIT	10 weeks, 3 days/week, sSIT: 4 × 5 reps, 5 sec all-out running; 4 × 6 reps, 5 sec all-out running; 4 × 7 reps, 5 sec all-out running; 4 × 8 reps, 5 sec all-out running; RT: 3 × 12 RM, ~ 70%; 3 × 10 RM, ~ 75%; 3 × 8 RM, ~ 80%; 3 × 6 RM, ~85%.	Countermovement vertical jump (VERTEC); 20-m sprint (photocell gates); VO_2_max (breath-by-breath gas collection system)	Countermovement vertical jump↑; 20-m sprint↓; VO_2_max↑
Zhang et al. (27)	China; RCT	*n* = 48; sex (male); EG1 (8) age = 19.7 ± 0.7 years; EG2 (8) age = 19.8 ± 0.7 years; CON (8) age = 20.1 ± 0.8 years competitive level: regional	judo	HIIT	6weeks, 2 days/week, EG1: [2 × 9 × (20:10 s) × (≥85% HRmax)]/2.5 min Total work time: 6 min; EG2: [2 × 6 × (30:10 s) × (≥85% HRmax)]/2.5 min Total work time: 6 min.	30-m sprint test (wireless photocell gates); Countermovement jump testvertical jump mat (vertical jump mat); Medicine ball put test (Meter stick); VO_2_max (20-m multistage shuttle run test)	30-m sprint test↓; Countermovement jump testvertical jump mat↑; Medicine ball put test↑; VO_2_max↑
Ojeda-Aravena et al. (28)	Chile; RCT	*n* = 10; sex (male, female); EG (5) age = 16.1 ± 1.12 years; CON (5) age = 14.5 ± 2.0 years competitive level: national	karate	HIIT	4 weeks, 3 days/week, the HIIT intervals were based on the documented temporal structure for this sport (1:2).	Jumping ability (electronic contact platform); change of direction speed (*t*-test)	Jumping Ability↑; Change of Direction Speed↓
Ojeda-Aravena et al. (29)	Chile; RCT	*n* = 12; sex (male, female); EG (6) age = 16.8 ± 2.5 years; CON (6) age = 17.8 ± 3.8 years competitive level: national	Taekwondo	HIIT	4 weeks, 3 days/week, three rounds of 2 min of activity (4 s of repeated efforts with 28 s of active pause; effort/pause ratio of 1:7) with 1 min of passive pause between rounds.	Jumping ability (electronic contact platform); aerobic Fitness (20-m shuttle run)	Jumping Ability↑; Aerobic Fitness↑
Ojeda-Aravena et al. (30)	Chile; RCT	*n* = 16; sex (male, female); EG (8) age = 17.4 ± 2.9; CON (8) age = 20.5 ± 5.0 years competitive level: national	Taekwondo	HIIT	4 weeks, 3 days/week, the EG performed a HIIT programme with 4 s of effort followed by 28 s of pause (effort: pause ratio 1:7) using alternating roundhouse kicks with both legs at maximum intensity (i.e., all-out) considering an RPE of 10 in front of a partner.	Jump ability (electronic contact platform); linear sprint in 5 m (electrical photocells); 20-metre shuttle run test (electrical photocells); anthropometric and body composition assessments	Jumping Ability↑; 20-m sprint↓; fat mass percentage↓; fat mass percentage↓; body mass↓
Franchini et al. (31)	Brazil; RCT	*n* = 35; sex (male, female); EG1 (9) age = 12 ± 7 years; EG2 (9) age = 15 ± 7 years; EG3 (9) age = 12 ± 7 years; CON (8) age = 18 ± 7 years competitive level: regional	Judo	HIIT	4 weeks, 2 days/week, the training session lasted for a total of 22 min 40 s, taking into account the 5-min warm-up, 3-min rest after warm-up, one block of 4 min 50 s of high-intensity intermittent exercise (ten times 20 s effort by 10 s pause), 5-min recovery and the 2nd block of high-intensity intermittent workout.	VO_2_max (20-m multistage shuttle run test); HRmax (polar H10 heart rate monitor)	VO_2_max↑; HRmax↓
Zhao et al. (32)	China; RCT	*n* = 30; sex (male); EG (15) age = 15.4 ± 1.68; CON (15) age = 16.3 ± 2.02 years competitive level: regional	Sanda	HIIT	6 weeks, 3 days/week, 6–12 sets of training, including 30 seconds of repeated 10-meter sprints, 60 seconds of specialized technical combination shooting, and 120 seconds of double-swinging rope skipping with explosive power, with a 30-s break between sets and a 3-minute break between each event.	Speed 4(Infrared laser system); Jump Ability (Three-dimensional force measurement platform)	Jump Ability↑; Speed↓
Wang (33)	China; RCT	*n* = 16; sex (male); EG (8) age = 22.1 ± 3.2; CON (8) age = 21.6 ± 3.5 years competitive level: regional	Wrestling	HIIT	8 weeks, 3 days/week, maximal sprint training (6 × 30m, 20s rest) was followed by cyclic power circuits of six explosive movements (30s each, 5 sets, 60s rest), high-speed weighted cloth man bridge throws (bodyweight load, 30s × 5 sets, 60s rest), and rapid unloaded wrestling technique drills (four techniques, 30s each, 5 sets, 60s rest between movement blocks).	HRmax (Real-time heart rate telemetry system); body composition (inbody3.0 body composition analyzer)	HRmax↑; Weight↓; percent body fat↓
Song & Sheykhlouvand (34)	China; RCT	*n* = 30; sex (male); age = 19.8 ± 1.3 years; competitive level: regional	Taekwondo	HIIT	6 weeks, 3 days/week, participants of HIITTS completed 3 sets of 10 × 4 s all-out repeated kicks with both legs, with 15 s passive recovery between efforts and 1 m rest between sets. HIITRS completed the same sets and repetitions as HIITTS but all-out running instead of repeated kicks.	VO_2_max (treadmill); vertical jump (globus electronic contact mat system); 20-m sprint (Stopwatch); agility (electronic timing system)	VO_2_max↑; Vertical jump↑; 20-m sprint↓; agility↓
Xu et al. (35)	China; RCT	*n* = 30; sex (male, female); EG1 (10) age = 16.80 ± 1.40 years; EG2 (10) age = 16.60 ± 1.43 years; CON (10) age = 17.60 ± 1.07 years competitive level: regional	Taekwondo	HIIT	6 weeks, 3 days/week, the HIIT-20S protocol involved four sets per round at a 20:10 s work-to-rest ratio, whereas the HIIT-30S protocol involved three sets per round at a 30:10 s work-to-rest ratio.	Agility (improved illinois agility test); lower limbs strength (1-RM squat test)	Lower limbs strength↓; Agility↓
Ouergui et al. (36)	Tunisia; RCT	*n* = 36; sex (male, female); age= 16 ±1 years; competitive level: regional	Taekwondo	RST RTT	4 weeks, 2 days/week, RST: 10 × 35 m sprint running with 10 s of passive rest between repetitions. RTT: completed 10 × 6 s as many repetitions as possible of a taekwondo technique, intercepted with 10 s of passive rest between series.	VO_2_max (20 m multistage shuttle run test); Speed (5 m shuttle run test); Jump height (countermovement jump); Agility (t-test)	VO_2_max↑; Speed↓; Jump height↑; Agility↓
Huang et al. (37)	Tunisia; RCT	*n* = 12; sex (male); EG (6) age = 20.83 ± 0.60; CON (6) age = 20.67 ± 0.59 years competitive level: regional	Taekwondo	HIIT	4 weeks, 5 days/week, Each technique was performed for 21 s, followed by 12 s of rest (WRR: 1.75:1). A resting period of 1 min was ensured between two rounds. in this phase, the participants performed their kicks at 85% of their HRmax.	HRmax (Polar HR monitor)	HRmax↓
Lee et al. (38)	Korea; RCT	*n* = 19; sex (male, female); EG (10) age = 20.83 ± 0.60; CON (9) age = 20.67 ± 0.59 years competitive level: national	Judo	ITP	12 weeks, 4 days/week, Intensive interval training was conducted at 80% maximal aerobic velocity (MAV), which was determined via VO2max test during first 2 weeks and at 90% MAV from week 3 to week 12.	VO_2_max (treadmill); HRmax (treadmill); body composition (DSM-BIA)	VO_2_max↑; HRmax↓; Percent body↓; Weight↓

ITP, intermittent training program; PBF, percent body fat; SMM, skeletal muscle ass; BM, body mass; RT, Resistance training; SIT, sprint interval training; HIIT, high-intensity interval training.

Table 1 summarizes the key characteristics of the included studies. The table provides detailed information on the countries of the studies, the specific contents of the intervention methods, outcome measures, and their measurement techniques. The majority of studies employed HIIT as the form of activity. The duration of the interventions varied across studies, ranging from 4 to 12 weeks. Such a wide variation in intervention duration is noteworthy, as a 4-week intervention may be insufficient to induce meaningful physiological adaptations, particularly in trained athletes. These differences in intervention designs could contribute to the variability in the reported outcomes, highlighting the methodological heterogeneity in the current literature. This variability may reflect different strategies employed to assess the outcomes of aerobic capacity, physical fitness, and body composition in martial arts athletes through HIIT.

### 3.3 Methodological quality of included studies

**Figure 3:**
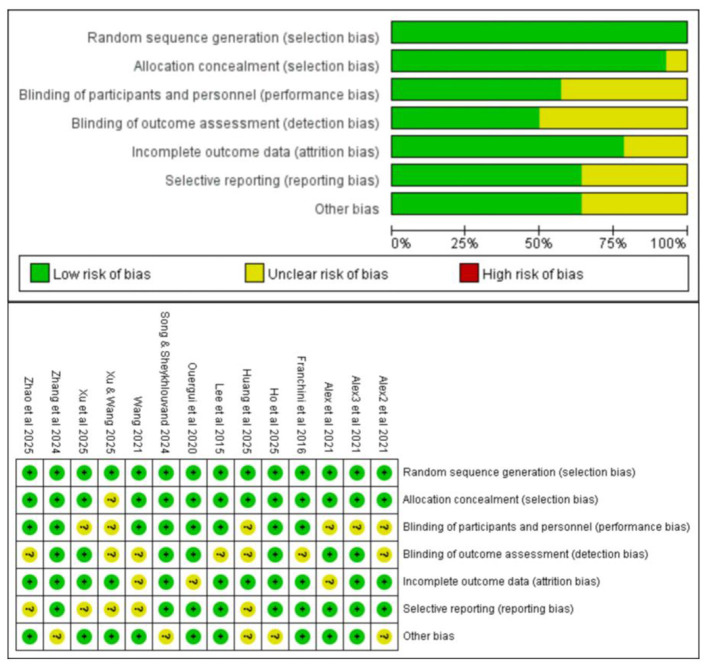
Risk of bias graph and Risk of bias summary.

The quality of the included studies was assessed using the Cochrane risk of bias tool, with the results shown in Figure 3. The quality assessment covers seven domains, and the results show that most studies had a low risk of bias in “random sequence generation” (selection bias) and “blinding of outcome assessment” (detection bias), marked in green. However, a few studies exhibited higher risks in “selective reporting” (reporting bias) and “incomplete outcome data” (attrition bias), marked in yellow, indicating uncertain or clear risks of bias in these areas. Overall, most studies performed well in “allocation concealment” and “blinding of participants and personnel”, but some studies still showed some degree of risk uncertainty. This result helps to systematically identify potential sources of bias and provides methodological guidance for the interpretation of subsequent meta-analysis results.

### 3.4 Effects of HIIT on aerobic capacity in martial arts athletes

**Figure 4:**
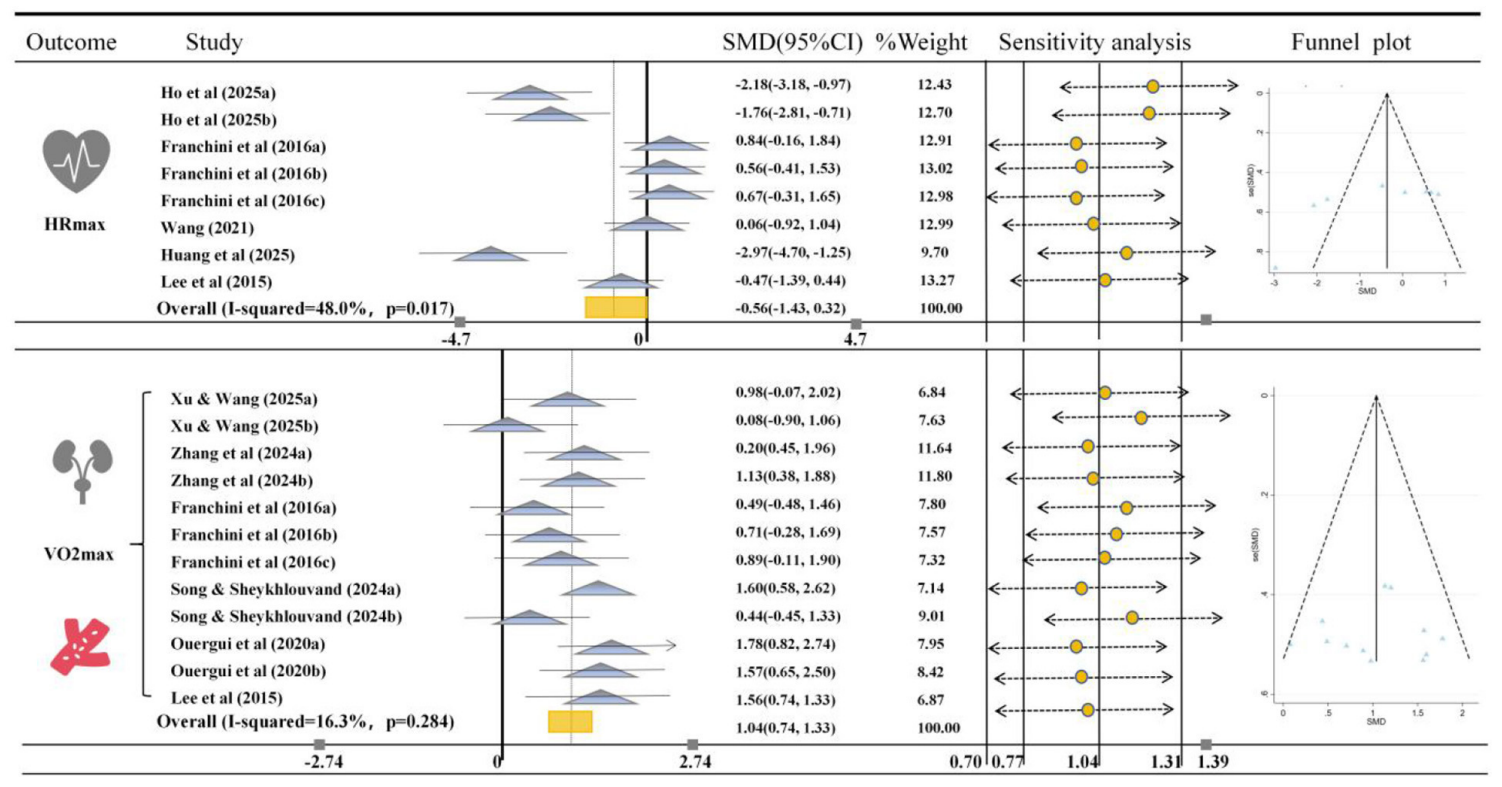
Meta-analysis of the effects of HIIT on HRmax and VO_2_max in Martial Arts Athletes. The figure presents the results of a meta-analysis comparing the effects of HIIT on HRmax and VO_2_max in martial arts athletes. The SMD (standardized mean difference) with 95% confidence intervals (CIs) for each study is displayed. The rectangle markers indicate the overall effect size, and the I^2^statistic shows the heterogeneity of each outcome.

Figure 4 analyzes the impact of HIIT on the aerobic capacity of martial arts athletes. Regarding HRmax, 8 studies provided relevant data comparing the effects of HIIT with conventional training or other non-HIIT interventions. The results showed that HIIT did not result in significant improvements in HRmax for martial arts athletes (*SMD* = −0.56, 95% CI [−1.43, 0.32], *I*^2^ = 48%, *p* = 0.017). It is important to note that a reduction in HRmax does not necessarily indicate a lack of improvement, in some cases, a decrease in HRmax can reflect improved cardiovascular efficiency and adaptation, as the heart becomes more efficient at delivering oxygen to the muscles during exercise. Thus, the interpretation of HRmax changes should be considered within the context of individual physiological adaptations. Additionally, the heterogeneity of the results was low, the funnel plot showed symmetry, and sensitivity analysis confirmed the robustness of the results.

For VO_2_max, 12 studies provided relevant data comparing the effects of HIIT with conventional training or other non-HIIT interventions. The results showed that HIIT significantly improved VO_2_max in martial arts athletes (*SMD* = 1.04, 95% CI [0.74, 1.33], *I*^2^ = 16.3%, *p* = 0.284). The heterogeneity of this result was low, and the funnel plot results, along with sensitivity analysis, confirmed the robustness of the study (Figure 4).

### 3.5 Effects of HIIT on physical fitness in martial arts athletes

**Figure 5:**
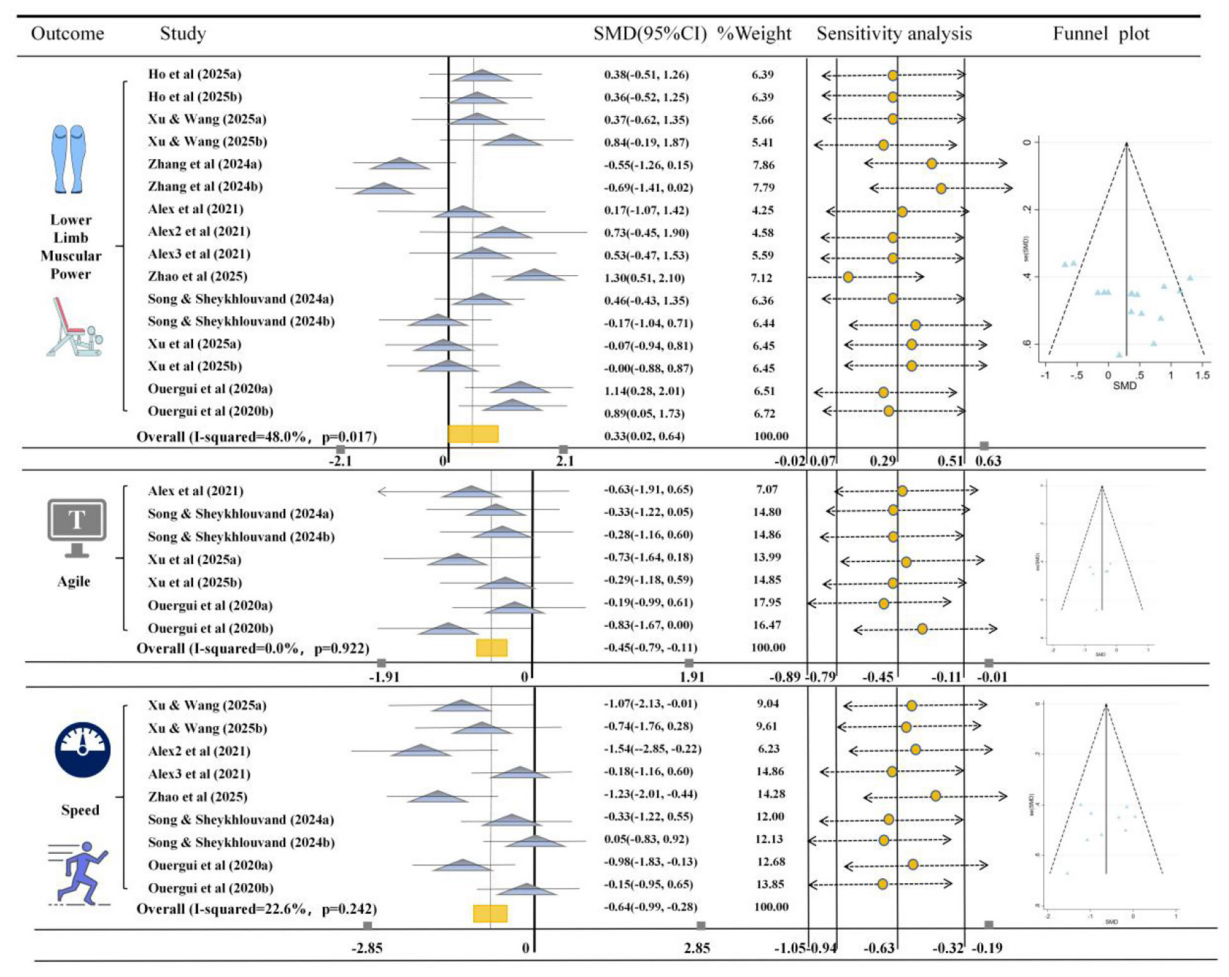
Meta-analysis of the effects of HIIT on lower limb muscular power, agility, and speed in martial arts athletes. The figure presents the results of a meta-analysis comparing the effects of HIIT on lower limb muscular power, agility, and speed in martial arts athletes. The SMD (standardized mean difference) with 95% confidence intervals (CIs) for each study is displayed. The rectangle markers indicate the overall effect size, and the *I*^2^statistic shows the heterogeneity of each outcome. Increases in lower limb muscular power reflect positive performance improvements, while decreases in agility and speed (likely expressed as time-based measures) should be interpreted as performance improvements due to faster times or more efficient movements.

Figure 5 analyzes the impact of HIIT on the physical fitness of martial arts athletes. For lower limb muscular power, 16 studies provided relevant data, comparing the effects of HIIT with conventional training or other non-HIIT interventions. The results showed that HIIT significantly improved lower limb muscular power in martial arts athletes (*SMD* = 0.33, 95% CI [0.02, 0.64], *I*^2^ = 48%, *p* = 0.017). Additionally, the heterogeneity of the result was low, the funnel plot showed symmetry, and sensitivity analysis confirmed the robustness of the results.

For agility, 7 studies provided relevant data, and the results showed that HIIT significantly improved agility in martial arts athletes (*SMD* = −0.45, 95% CI [−0.79, −0.11], *I*^2^ = 0%, *p* = 0.922). Similarly, the heterogeneity was low, and both the funnel plot and sensitivity analysis confirmed the robustness of the study results (Figure 5).

For speed, the results from 9 studies showed that HIIT also significantly improved agility in martial arts athletes (*SMD* = −0.64, 95% CI [−0.99, −0.28], *I*^2^ = 22.6%, *p* = 0.242). The heterogeneity was low, and both the funnel plot and sensitivity analysis confirmed the robustness of the study results (Figure 5).

### 3.6 Effects of HIIT on body composition in martial arts athletes

**Figure 6:**
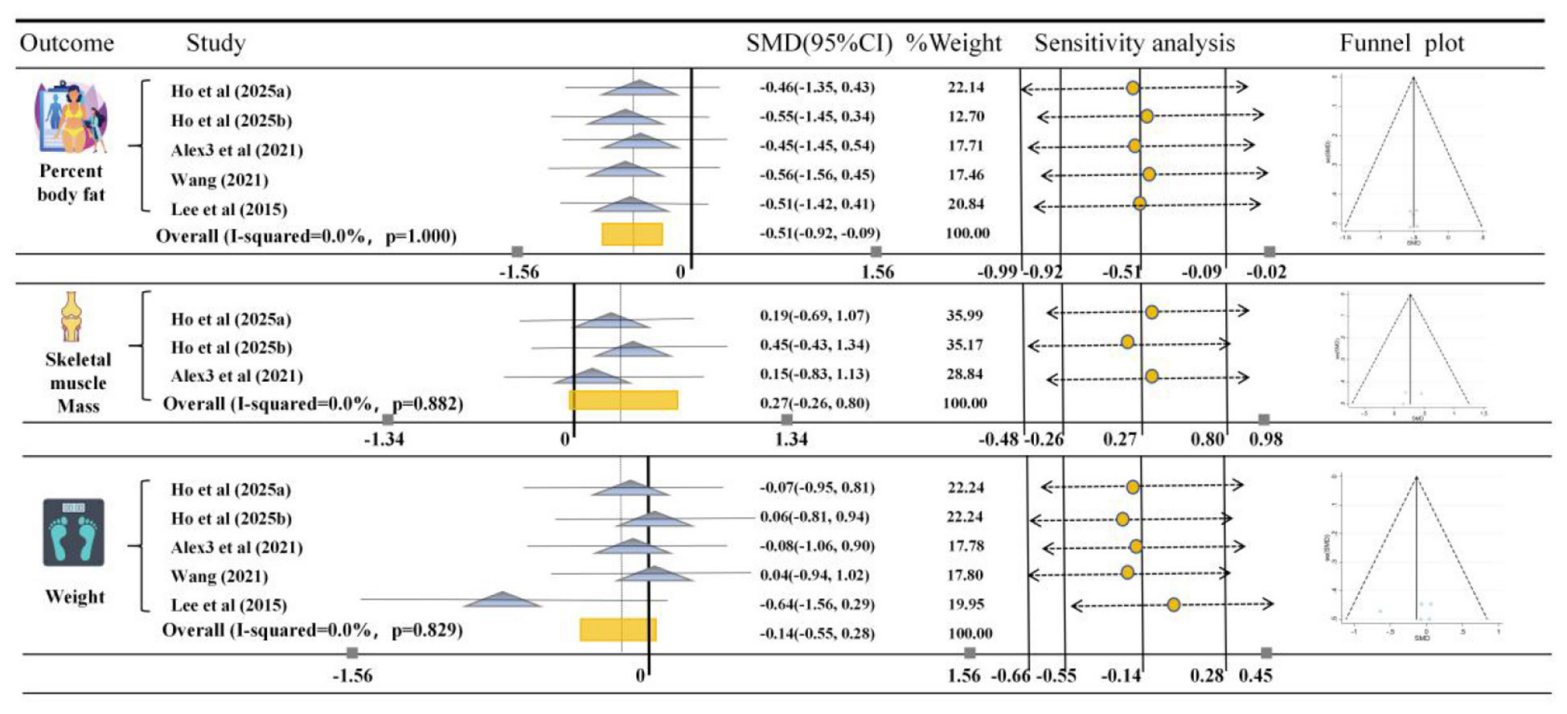
Meta-analysis of the effects of HIIT on Percent body fat, Skeletal muscle mass, and Weight in Martial Arts Athletes. The figure presents the results of a meta-analysis comparing the effects of HIIT on Percent body fat, Skeletal muscle mass, and Weight in martial arts athletes. The SMD (standardized mean difference) with 95% confidence intervals (CIs) for each study is displayed. The rectangle markers indicate the overall effect size, and the *I*^2^statistic shows the heterogeneity of each outcome.

The results of Figure 6 show that HIIT has a significant improvement effect on percent body fat in martial artists (*SMD* = −0.51, 95% CI [−0.92, −0.09], *I*^2^ = 0%, *p* = 1.000), but no significant improvement was observed for skeletal muscle mass (*SMD* = 0.27, 95% CI [−0.26, 0.80], *I*^2^ = 0%; *p* = 0.882) and weight (*SMD* = −0.14, 95% CI [−0.55, 0.28], *I*^2^ = 0%; *p* = 0.829). Low heterogeneity, sensitivity, and funnel plots all confirm the robustness of the study.

### 3.7 Publication bias and sensitivity analysis

In (Figures 4–6), publication bias analysis showed no significant risk, indicating that the results are relatively robust. Similarly, we conducted a sensitivity analysis, excluding individual studies that had minimal impact on the overall effect estimate, suggesting that this meta-analysis demonstrates high consistency and reliability. Subsequently, an Egger test was performed, and the results showed (Supplementary Table S2) that HRmax (*p* = 0.088), VO_2_max (*p* = 0.879), lower limb muscular power (*p* = 0.133), agility (*p* = 0.579), speed (*p* = 0.413), percent body fat (*p* = 0.967), skeletal muscle mass (*p* = 0.682), and weight (*p* = 0.930) did not show significant publication bias, further supporting the robustness of the study results. Therefore, the overall analysis results remain reliable and robust.

## 4 Discussion

This study explored the effects of HIIT on the aerobic capacity, physical fitness, and body composition of martial arts athletes through meta-analysis and systematic review. To enhance the quality of evidence and the reliability of conclusions, this analysis was strictly limited to randomized controlled trials. The results show that HIIT led to significant improvements in several key indicators, including VO_2_max, lower limb muscular power, agility, speed, and percent body fat. However, no statistically significant changes were observed in HRmax, skeletal muscle mass, and weight. The duration of the interventions varied across studies, ranging from 4 to 12 weeks. Such a wide variation in intervention duration is noteworthy, as interventions lasting only 4 weeks may be insufficient to induce meaningful physiological adaptations, particularly in trained athletes. These differences in intervention designs could contribute to the variability in the reported outcomes, highlighting the methodological heterogeneity in the current literature. This variability may reflect different strategies employed to assess the outcomes of aerobic capacity, physical fitness, and body composition in martial arts athletes through HIIT. It is important to note that the lack of significant changes in HRmax, skeletal muscle mass, and weight may be partly due to the relatively short duration of some interventions, especially those lasting only 4 weeks. Such a short intervention period is likely insufficient to induce measurable adaptations in aerobic capacity, performance, or body composition, particularly in trained athletes. Therefore, future studies should consider longer intervention periods or the integration of HIIT with other training methods to achieve more comprehensive and sustainable improvements. These findings suggest that while HIIT can significantly enhance endurance, explosiveness, agility, and body composition, its effects on certain physiological and morphological indicators may require longer durations or additional training strategies for more substantial changes.

This study found that HIIT significantly improved the VO_2_max of martial artists, a finding consistent with previous research on HIIT for athletes in other sports (39–41). For martial artists, their movements are predominantly high-intensity and intermittent, but the continuous offensive-defensive transitions and multi-round competition format impose high demands on cardiovascular function (42, 43). HIIT, through repeated high-intensity stimuli or near-maximal oxygen uptake intensity, can effectively enhance myocardial contractility and capillary density, thereby improving oxygen delivery and utilization efficiency. It is noteworthy that while VO_2_max significantly improved, HRmax did not increase concurrently, which differs from previous studies (44, 45). This may suggest that the cardiovascular adaptations induced by HIIT are more focused on optimizing stroke volume and oxygen extraction efficiency rather than solely relying on heart rate compensation, reflecting an effective improvement in cardiac function to some extent (46, 47).

In this study, the significant improvements in lower limb muscle strength, agility, and speed directly demonstrate the positive impact of HIIT on martial arts-specific performance. Whether in Taekwondo, Karate, or other martial arts, the characteristics of technical movements rely on the ability to output high power in a short time (48, 49), while the high-intensity phases in HIIT effectively recruit fast-twitch muscle fibers (50) and enhance neuromuscular coordination and force production rate (51). Furthermore, HIIT often includes multidirectional speed changes and explosive movements, which align well with the demands for quick direction changes, dodging, and continuous attacks in martial arts, possibly explaining the concurrent improvement in agility and speed.

In addition, this study found that HIIT significantly reduced body fat percentage in martial artists, but had no significant effect on skeletal muscle mass and body weight. This is consistent with previous research (52), which indicates that although HIIT can effectively mobilize lipid metabolism and increase post-exercise excess oxygen consumption, its effect on promoting muscle hypertrophy may be weaker than that of traditional resistance training (53, 54). Martial artists often need to maintain strength while controlling their body weight; therefore, a simple HIIT intervention may not be sufficient to achieve significant increases in muscle mass.

### 4.1 Limitations and future prospects

Although the results of this study confirm the effective impact of HIIT on martial arts athletes, several limitations remain. Firstly, the small sample size of the RCTs included in this study may affect the statistical power of the results. Secondly, there are significant differences across studies in the HIIT protocols (e.g., intensity, frequency, duration, and structure), participants' training backgrounds, and measurement tools, which may have contributed to substantial heterogeneity in the results. Treating HIIT as a single, homogeneous intervention overlooks the potential variations in training protocols, which could significantly influence the pooled outcomes and complicate the interpretation of the findings. Furthermore, there are few long-term intervention studies among the included research, and the sustainability of HIIT effects remains unclear. Future research should focus on conducting more large-sample, long-term, and standardized randomized controlled trials, with further attention to the differential responses in martial arts athletes of different genders and levels.

Despite the limitations of this study, it still provides important evidence for the scientific training of martial arts athletes. In practical applications, coaches can base their approach on existing evidence, using HIIT as an effective supplement to improve athletes aerobic capacity, explosive power, agility, and body composition, especially in pre-competition intensification phases or time-limited training scenarios. Future studies are recommended to further explore the optimal integration of high-intensity interval training with martial arts-specific technical training, and to systematically incorporate physiological and biochemical indicators to scientifically monitor training load and recovery processes. Additionally, it is necessary to conduct comparative studies across combat sports to clarify the differential effects of high-intensity interval training in various martial arts.

## 5 Conclusion

This systematic review and meta-analysis suggests that HIIT can significantly improve martial artists' VO_2_max, lower limb muscular power, agility, speed, and percent body fat, but no significant improvements were found in HRmax, skeletal muscle mass, and weight. It is important to note that existing evidence still has certain limitations, including small sample sizes in the included studies, heterogeneity in intervention protocols and assessment methods, and a lack of long-term follow-up data. Therefore, future research should focus on conducting more large-scale, high-quality, long-term randomized controlled trials to further validate the sustained benefits of HIIT and the best practice models in martial artist training.

## Data Availability

The original contributions presented in the study are included in the article/Supplementary material, further inquiries can be directed to the corresponding authors.
