# Supplementary material for: Effect of high-intensity interval training on aerobic capacity, physical fitness, and body composition in martial arts athletes: a systematic review and meta-analysis of randomized controlled trials
Source: Front Nutr. 2026 Mar 31;13:1792680. doi: 10.3389/fnut.2026.1792680 (PMC13076352; doi:10.3389/fnut.2026.1792680)

**Table S1** Search strategy

| Data | Query | Results |
| --- | --- | --- |
| Pubmed | ((((((((((((((((high-intensity interval training[Title/Abstract]) OR (HIIT[Title/Abstract])) OR (intermittent exercise[Title/Abstract])) OR (sprint interval training[Title/Abstract])) OR (repeated sprint training[Title/Abstract])) AND (Martial Arts[Title/Abstract])) OR (Judo[Title/Abstract])) OR (Karate[Title/Abstract])) OR (Kung Fu[Title/Abstract])) OR (Gongfu[Title/Abstract])) OR (Gong Fu[Title/Abstract])) OR (Tae Kwon Do[Title/Abstract])) OR (Wushu[Title/Abstract])) OR (Hap Ki Do[Title/Abstract])) OR (Aikido[Title/Abstract])) OR (Jujitsu[Title/Abstract])) AND (Athletes[Title/Abstract]) | 1007 |
| Web of science | ((((((((((((((((((TS=(High-Intensity Interval Training)) OR TS=(hint)) OR TS=(High Intensity Interval Training)) OR TS=(High-Intensity Interval Trainings)) OR TS=(High-Intensity Intermittent Exercise)) OR TS=(High-Intensity Intermittent Exercises)) OR TS=(Sprint Interval Trainings)) AND TS=(Martial Arts)) OR TS=(Judo)) OR TS=(Karate)) OR TS=(Kung Fu)) OR TS=(gonghu)) OR TS=(Gong Fu)) OR TS=(Tae Kwon Do)) OR TS=(washu)) OR TS=(Hap Ki Do)) OR TS=(anilido)) OR TS=(jujutsu)) AND TS=(Athletes) | 1349 |
| MEDLINE | ((((((((((((((((((TS=(High-Intensity Interval Training)) OR TS=(hint)) OR TS=(High Intensity Interval Training)) OR TS=(High-Intensity Interval Trainings)) OR TS=(High-Intensity Intermittent Exercise)) OR TS=(High-Intensity Intermittent Exercises)) OR TS=(Sprint Interval Trainings)) AND TS=(Martial Arts)) OR TS=(Judo)) OR TS=(Karate)) OR TS=(Kung Fu)) OR TS=(gonghu)) OR TS=(Gong Fu)) OR TS=(Tae Kwon Do)) OR TS=(washu)) OR TS=(Hap Ki Do)) OR TS=(anilido)) OR TS=(jujutsu)) AND TS=(Athletes) | 1169 |
| ProQuest | ((((((((((((((((((TS=(High-Intensity Interval Training)) OR TS=(hint)) OR TS=(High Intensity Interval Training)) OR TS=(High-Intensity Interval Trainings)) OR TS=(High-Intensity Intermittent Exercise)) OR TS=(High-Intensity Intermittent Exercises)) OR TS=(Sprint Interval Trainings)) AND TS=(Martial Arts)) OR TS=(Judo)) OR TS=(Karate)) OR TS=(Kung Fu)) OR TS=(gonghu)) OR TS=(Gong Fu)) OR TS=(Tae Kwon Do)) OR TS=(washu)) OR TS=(Hap Ki Do)) OR TS=(anilido)) OR TS=(jujutsu)) AND TS=(Athletes) | 157 |
| SciELO | ((((((((((((((((((TS=(High-Intensity Interval Training)) OR TS=(hint)) OR TS=(High Intensity Interval Training)) OR TS=(High-Intensity Interval Trainings)) OR TS=(High-Intensity Intermittent Exercise)) OR TS=(High-Intensity Intermittent Exercises)) OR TS=(Sprint Interval Trainings)) AND TS=(Martial Arts)) OR TS=(Judo)) OR TS=(Karate)) OR TS=(Kung Fu)) OR TS=(gonghu)) OR TS=(Gong Fu)) OR TS=(Tae Kwon Do)) OR TS=(washu)) OR TS=(Hap Ki Do)) OR TS=(anilido)) OR TS=(jujutsu)) AND TS=(Athletes) | 156 |
| CNIK | （主题：高强度间歇运动）OR （主题：HIIT）AND （主题：空手道）OR （主题：跆拳道） AND （主题：运动员） | 770 |

**Table S2** Egger's test

(lower limb muscular power)


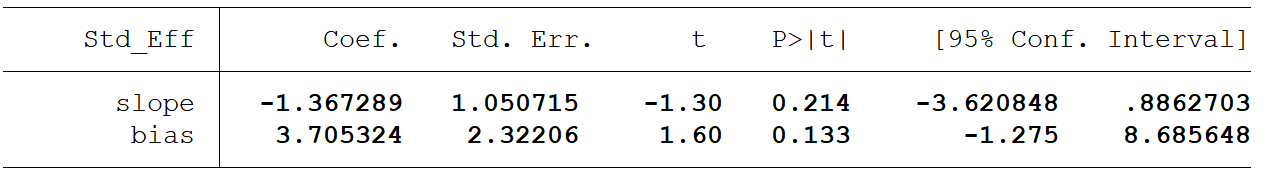


(agile)


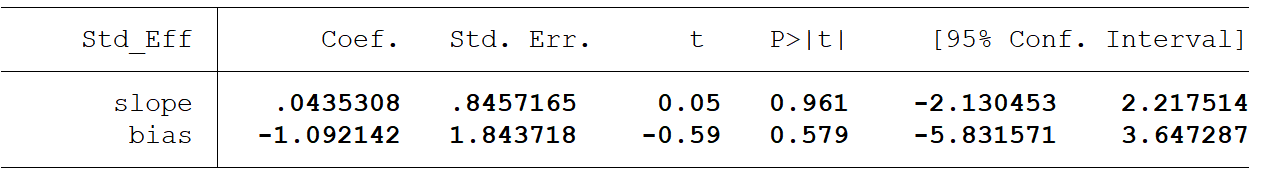


(speed)


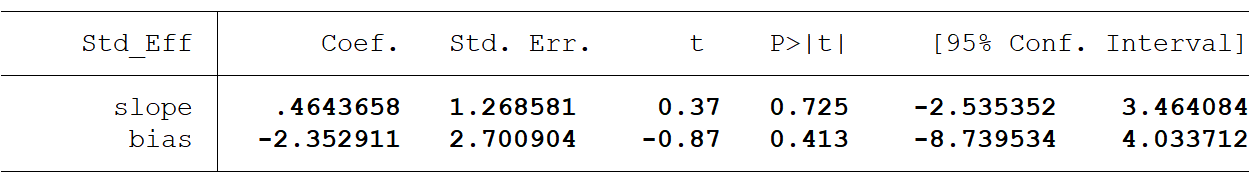


(HRmax)


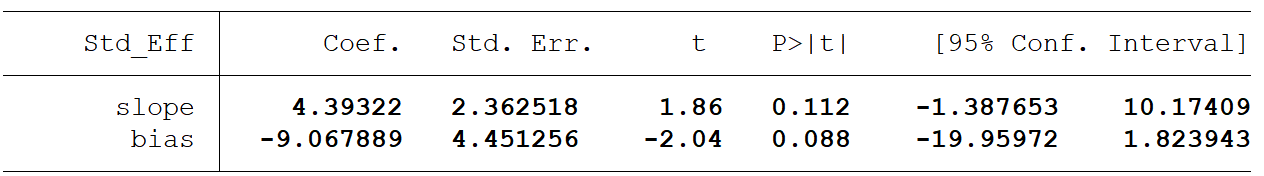


(VO2max)


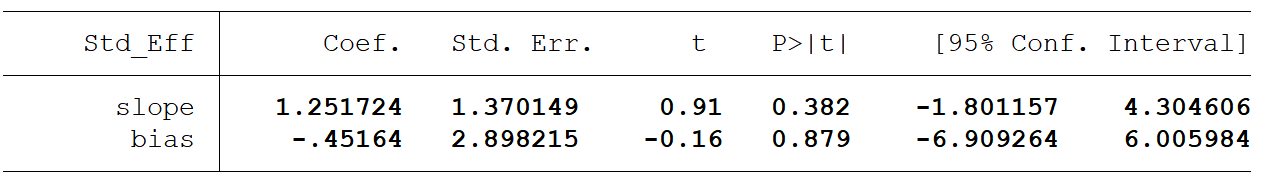


(Percent body fat)


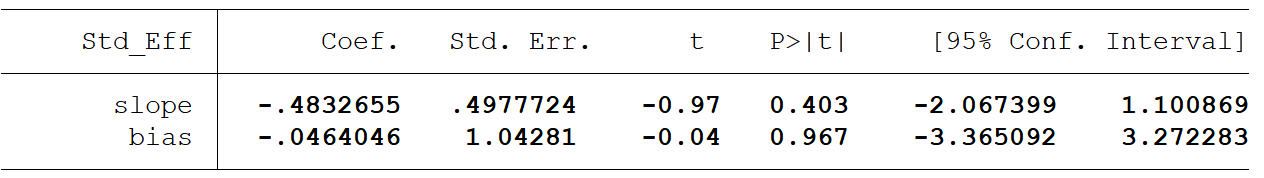


(skeletal muscle mass)


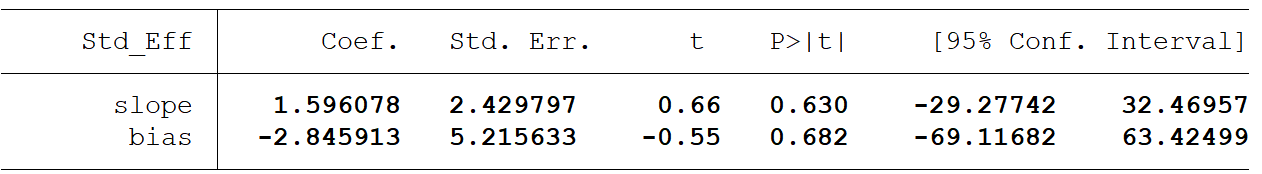


(weight)


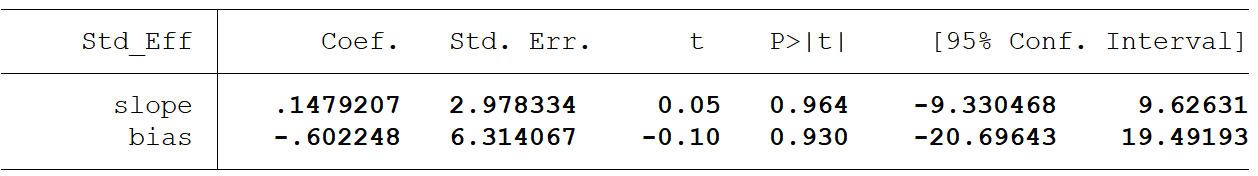

Supplement: Supplementary file 1 [file Supplementary_file_1.docx]
